# Supplementary material for: Quality control for single-cell analysis of high-plex tissue profiles using CyLinter
Source: Nat Methods. 2024 Oct 30;21(12):2248–59. doi: 10.1038/s41592-024-02328-0 (PMC11621021; doi:10.1038/s41592-024-02328-0)
Supplement: Supplementary file 1 — Supplementary Figs. 1–3, Table 1 and Notes 1 and 2, links to Online Supplementary Figs. 1–10 and links to lists of centers and principal investigators participating in the HTAN and HuBMAP consortia. [file 41592_2024_2328_MOESM1_ESM.pdf]

nature methods

Supplementary information https://doi.org/10.1038/s41592-024-02328-0

Quality control for single-cell analysis of high-plex tissue profiles using CyLinter

In the format provided by the authors and unedited

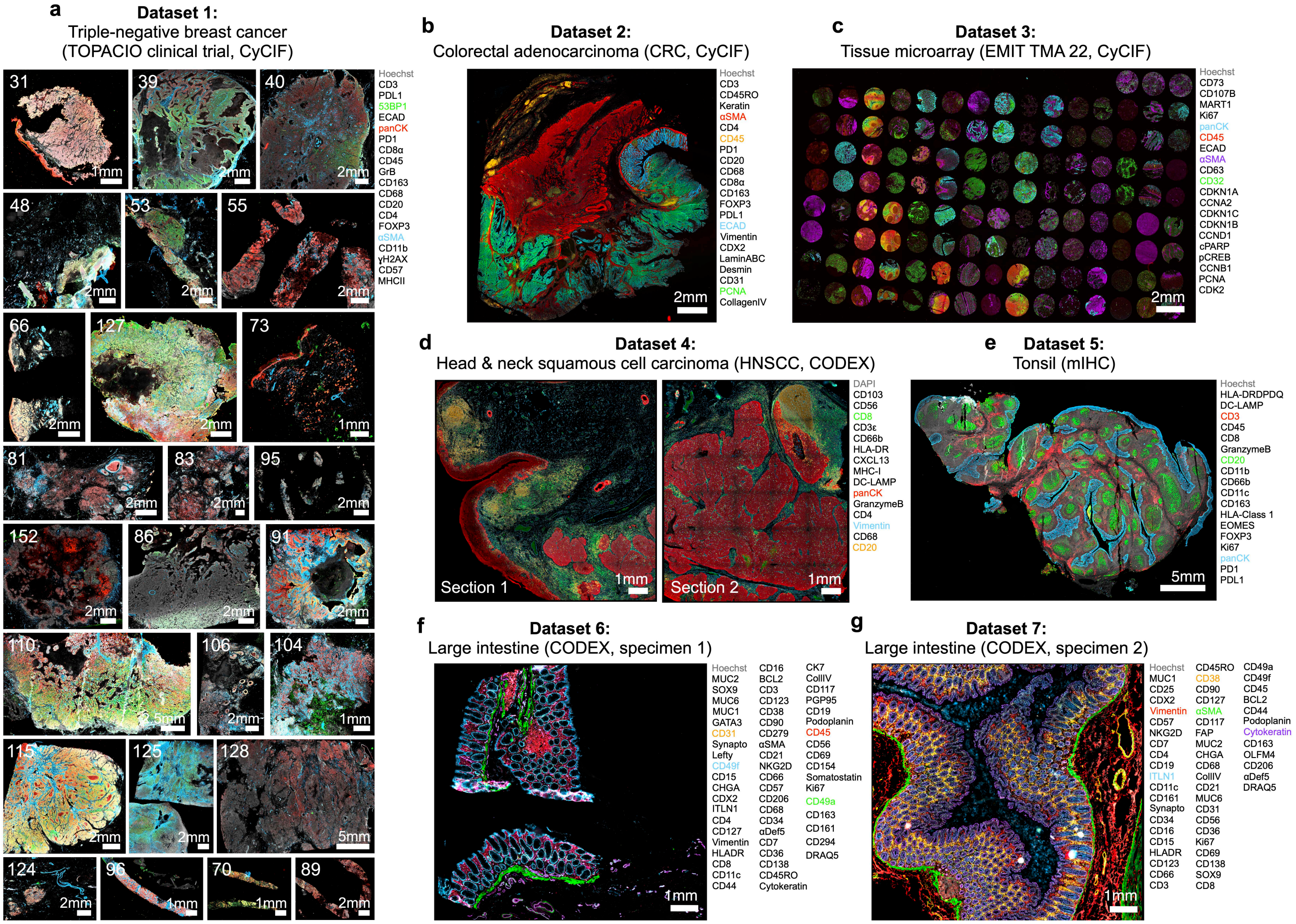

Supplementary Fig. 1 | Overview of the seven multiplex IF datasets analyzed in this study.
a, Dataset 1 (TOPACIO, CyCIF): 25 human TNBC clinical trial specimens (~6-353 mm^2). Numbers in upper left of each panel indicate specimen number. Channels shown are Hoechst (gray), 53BP1 (green), panCK (red), and αSMA (blue). **b**, Dataset 2 (CRC, CyCIF): an ~172 mm^2 whole-slide section of primary human colorectal adenocarcinoma. Channels shown are Hoechst (gray), αSMA (red), CD45 (orange), ECAD (blue), and PCNA (green). **c**, Dataset 3 (EMIT TMA22, CyCIF): 123 healthy and diseased human tissue cores each ~2 mm^2 arranged on a single microscope slide. Channels shown are Hoechst (gray), panCK (blue), CD45 (red), αSMA (purple), and CD32 (green). **d**, Dataset 4 (HNSCC, CODEX): two ~42 mm^2 whole-slide sections of human HNSCC. Channels shown are DAPI (gray), CD8 (green), panCK (red), vimentin (blue), and CD20 (orange). **e**, Dataset 5 (Tonsil, mIHC): an ~92 mm^2 whole-slide section of normal human tonsil. Channels shown are Hoechst (gray), CD3 (red), CD20 (green), panCK (blue). **f**, Dataset 6 (Large intestine, CODEX, specimen 1): an ~7 mm^2 whole-slide section of normal human large intestine. Channels shown are Hoechst (gray), CD31 (orange), CD49f (blue), CD45 (red), CD49a (green). **g**, Dataset 7 (Large intestine, CODEX, specimen 2): an ~12 mm^2 whole-slide section of normal human large intestine. Channels shown are Hoechst (gray), Vimentin (red), ITLN1 (blue), CD38 (orange), αSMA (green), Cytokeratin (purple). Markers to the right of each dataset indicate the full marker set for the corresponding image(s). See **Supplementary Table 1** for specimen metadata, identifiers, and data accession information.

Dataset	Data Type	Plex	# specimens	Image dimensions (x/y, pixels)	Nominal resolution (um/pixel) (idependent of effective optical resolution)	Tissue Area (mm2)	Source	Identifier	Data Access	Source publication DOI	Description
**1:** Human TNBC (triple-negative breast cancer)	CyCIF	20-plex	25	Range: (11239 x 8377) - (36060 x 26068)	0.65	Range: ~6-353	TOPACIO clinical Trial (ClinicalTrials.gov Identifier: NCT02657889); Vinayka et al. JAMA Oncol. 2019, PMCID: PMC6567845	N/A	Data can be released with explicit permission from the Clinical Trial Sponsor (Tesaro, Inc.)	10.1001/jamaoncol.2019.1029	25 fine-needle, punch-needle, or gross TNBC tissue biopsies (female, mixed age and ancestry)
**2:** Human colorectal adenocarcinoma (CRC)	CyCIF	22-plex	1	(26139 x 27120)	0.65	~172	Lin et al. Cell 2023, PMCID: PMC10019067	HTA13_1_101	HTAN portal (https://data.humantumoratlas.org/explore)	10.1016/j.cell.2022.12.028	Whole-slide section (1.6cm^2) of human colorectal adenocarcinoma tissue (69-year-old white male)
**3:** EMIT (Exemplar Microscopy Images of Tissues) TMA 22	CyCIF	21-plex	123	(2943 x 2943) per core	0.65	~2 (per core)	Schapiro et al. Nat. Methods 2022, PMCID: PMC8916956	syn22345750	Sage Synapse (https://www.synapse.org/#!Synapse:syn22345750)	10.1038/s41592-021-01308-y	123 healthy and cancerous human tissue cores (1.5mm diameter, 2-8 cores/tissue type; mixed age, sex, and ancestry)
**4:** Human head & neck squamous cell carcinoma (HNSCC)	CODEX	16-plex	2	section 1: (11900 x 11916), section 2: (10000 11800)	0.65	~42 (per specimen)	Laboratory of Kai Wucherpfennig	N/A	Dr. Kai Wucherpfennig, Dana-Farber Cancer Institute	N/A	2 whole-slide sections of a single HNSCC specimen (unknown origin)
**5:** Human tonsil	mIHC	19-plex	1	(29879 x 36539)	0.5	~92	Schapiro et al. Nat. Methods 2022, PMCID: PMC8916956	syn25174227	Sage Synapse (https://www.synapse.org/#!Synapse:syn25174227)	10.1038/s41592-021-01308-y	Whole-slide section of normal tonsil (4-year-old white female)
**6:** Human large intestine (sample 1)	CODEX	59-plex	1	(9995, 9515)	0.38	~7	HuBMAP data portal	HBM946.GRVG.379	HuBMAP data portal (https://portal.hubmapconsortium.org/browse/dataset/ae422532f260b3d6fc662aae69b05d33)	N/A	Whole-slide section of normal large intestine (78-year-old African American male)
**7:** Human large intestine (sample 2)	CODEX	54-plex	1	(9989 x 9505)	0.38	~12	HuBMAP data portal	HBM524.VWGB.378	HuBMAP data portal (https://portal.hubmapconsortium.org/browse/dataset/eaad67a6c6e891ea72cc397c26bd607f)	N/A	Whole-slide section of normal large intestine (24-year-old white male)

Supplementary Table 1 | Metadata, identifiers, and accession information for the seven multiplex IF datasets analyzed in this study. N/A = not available.

Supplementary Note 1

Impact of image background subtraction on tissue-derived, single-cell data.

Background subtraction is a common image processing technique used in immunofluorescence imaging to remove autofluorescence and fluorescence arising from non-specific antibody binding to a tissue. To improve antibody signal-to-noise, we applied rolling ball image background subtraction[1] to each channel of the TOPACIO dataset. However, we observed that when this was done the resulting histogram distributions of per cell channel intensities contained small numbers of zero-valued cells that resided far to the left of the bulk distribution (**Supplementary Fig. 2a**). These cells were also readily identified in the pre-QC TOPACIO UMAP embedding as extremely dim outliers occupying cell clusters at the extrema of the embedding space (**Supplementary Fig. 2b,c**). To better understand how these cells were distributed in the pre-QC TOPACIO clustering result, we highlighted where in the panCK signal distribution that cells from different clusters resided. Clusters within meta-cluster B (e.g., cluster 14) were exclusively composed of cells with zero-valued signals, while those in meta-cluster C (e.g., cluster 174) had signals all greater than zero, and those in meta-cluster F (e.g., cluster 197) were composed of a mixture of cells with zero and non-zero signals for panCK (**Supplementary Fig. 2d**).

As expected, the removal of zero-valued cells from the pre-QC TOPACIO dataset (in the absence of any other quality control measures) eliminated cells at the far left of the histogram distributions (**Supplementary Fig. 2e**). The cells in the UMAP embedding characterized by very low signal intensities were also eliminated when this was done, leading to a greater degree of resolution between the remaining immunopositive and immunonegative cell populations (**Supplementary Fig. 2f**). Further improvement in signal intensity resolution was observed after the additional removal of cells with near-zero signal intensities, which we presumed to also be affect by background subtraction, just to a lesser extent (**Supplementary Fig. 2g,h**). Scant numbers of extremely dim outliers were also observed in Dataset 6 (CODEX; **Supplementary Fig. 2i**), although the origin of these cells was less clear, as these data were not acquired by us, but rather previously acquired as part of the HuBMAP consortium. Nevertheless, removal of these cells also improved resolution in the signal intensity distributions of remaining cells (**Supplementary Fig. 2j**). Together we conclude that while background subtraction is useful for improving data quality by increasing antibody signal-to-noise, it can significantly skew the natural distribution of per cell channel signal intensities when it results in cells with signals at or near zero and can have a profound impact on the interpretation of single-cell data due to the formation of spurious cell clusters. We therefore suggest that when implementing image background subtraction, that one must be aware of and control for this potential confounder.

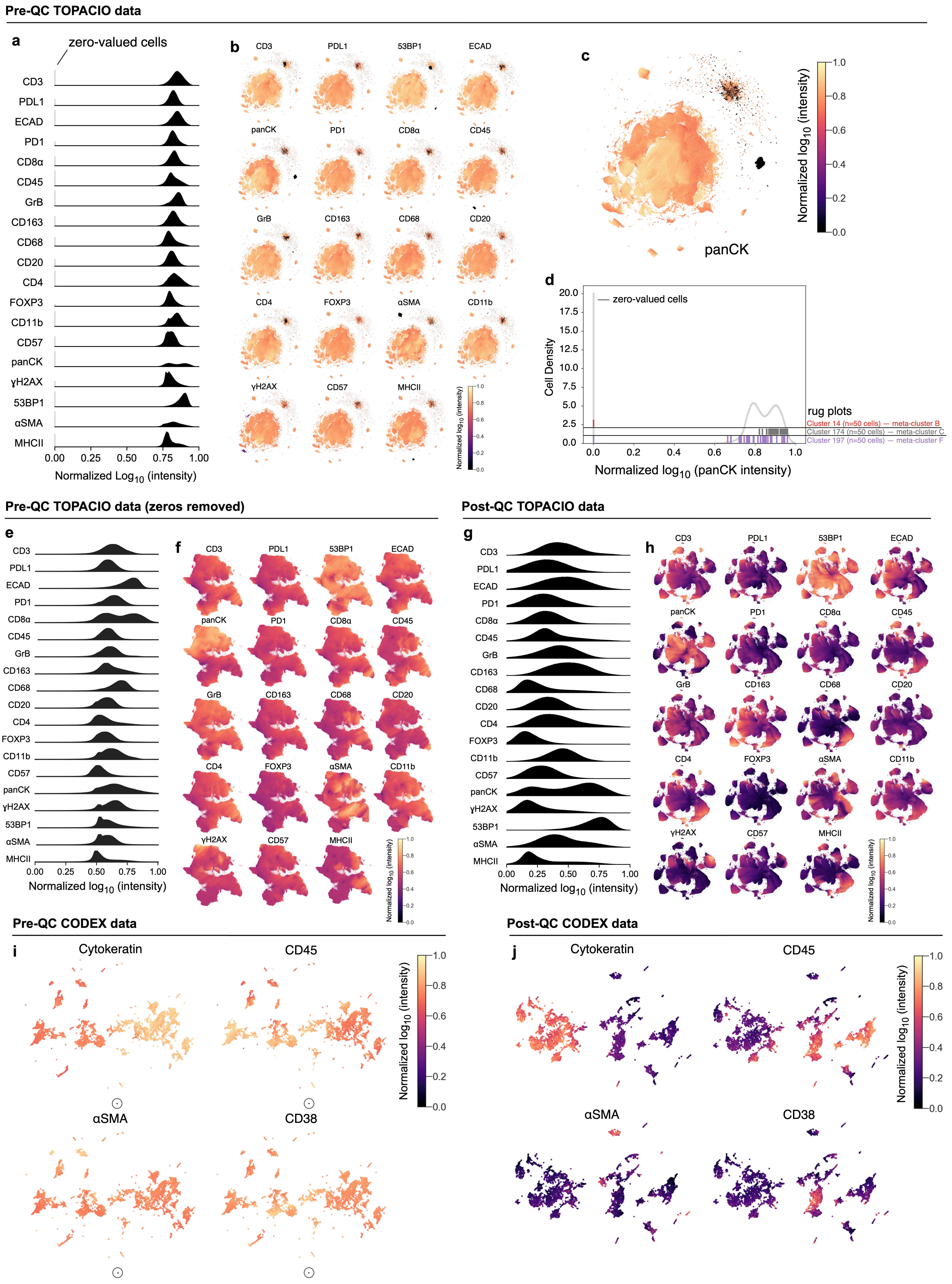
Pre-QC TOPACIO data
a
zero-valued cells
CD3
PDL1
ECAD
PD1
CD8α
CD45
GrB
CD163
CD68
CD20
CD4
FOXP3
CD11b
CD57
panCK
γH2AX
53BP1
αSMA
MHCII
0.00 0.25 0.50 0.75 1.00
Normalized Log10 (intensity)
b
CD3 PDL1 53BP1 ECAD
panCK PD1 CD8α CD45
GrB CD163 CD68 CD20
CD4 FOXP3 αSMA CD11b
γH2AX CD57 MHCII
c
panCK
Normalized log10 (intensity)
1.0 0.8 0.6 0.4 0.2 0.0
d
zero-valued cells
Cell Density
20.0 17.5 15.0 12.5 10.0 7.5 5.0 2.5 0.0
rug plots
Cluster 14 (n=50 cells) — meta-cluster B
Cluster 174 (n=50 cells) — meta-cluster C
Cluster 197 (n=50 cells) — meta-cluster F
0.0 0.2 0.4 0.6 0.8 1.0
Normalized log10 (panCK intensity)
Pre-QC TOPACIO data (zeros removed)
e
CD3
PDL1
ECAD
PD1
CD8α
CD45
GrB
CD163
CD68
CD20
CD4
FOXP3
CD11b
CD57
panCK
γH2AX
53BP1
αSMA
MHCII
0.00 0.25 0.50 0.75 1.00
Normalized log10 (intensity)
f
CD3 PDL1 53BP1 ECAD
panCK PD1 CD8α CD45
GrB CD163 CD68 CD20
CD4 FOXP3 αSMA CD11b
γH2AX CD57 MHCII
Post-QC TOPACIO data
g
CD3
PDL1
ECAD
PD1
CD8α
CD45
GrB
CD163
CD68
CD20
CD4
FOXP3
CD11b
CD57
panCK
γH2AX
53BP1
αSMA
MHCII
0.00 0.25 0.50 0.75 1.00
Normalized log10 (intensity)
h
CD3 PDL1 53BP1 ECAD
panCK PD1 CD8α CD45
GrB CD163 CD68 CD20
CD4 FOXP3 αSMA CD11b
γH2AX CD57 MHCII
Pre-QC CODEX data
i
Cytokeratin
CD45
αSMA
CD38
Normalized log10 (intensity)
1.0 0.8 0.6 0.4 0.2 0.0
Post-QC CODEX data
j
Cytokeratin
CD45
αSMA
CD38
Normalized log10 (intensity)
1.0 0.8 0.6 0.4 0.2 0.0

Supplementary Fig. 2 | Impact of image background subtraction on tissue-derived, single-cell data. a, Ridge plots showing the distribution of cells according to channel signal intensities in the pre-QC TOPACIO dataset which exhibit zero-valued cells at the far left of each distribution. **b**, Signal intensity colormaps applied to cells in the pre-QC TOPACIO embedding revealing the presence of extremely dim cells corresponding to those with zero-valued signal intensities, which by contrast makes all other cells in the embedding appear immunopositive for each marker. **c**, PanCK channel from panel (b) enlarged to show detail. **d**, Histogram distribution of cells in the pre-QC TOPACIO dataset according to their panCK signal (gray silhouette). Rugplot plots (vertical ticks) at bottom of histogram show where randomly selected cells from cluster 14 (meta-cluster B, red), cluster 174 (meta-cluster C, gray), and cluster 197 (meta-cluster F, purple) reside in the distribution (see **Fig. 3d** for meta-cluster assignments). **e**, Ridge plots showing the distribution of cells according to channel signal intensities in the pre-QC TOPACIO dataset after removal of zero-valued cells. **f,** Signal intensity colormaps applied to cells in the pre-QC TOPACIO embedding after removal of zero-valued cells exhibiting the absence of extremely dim cells and greater resolution in the signal intensities of remaining cells. **g**, Ridge plots showing the distribution of cells according to channel signal intensities in the post-QC TOPACIO dataset demonstrating a more natural distribution of signal intensities. **h,** Signal intensity colormaps applied to cells in the post-QC TOPACIO embedding showing high-contrast between immunopositive and immunonegative cell populations. **i**, Signal intensity colormaps applied to cells in the pre-QC Dataset 6 (CODEX) embedding exhibiting scant dim outliers (circles) that cause the rest of the cells to appear bright for each marker (see **Online Supplementary Fig. 9** for full set of UMAP plots colored by marker intensity). **j**, Signal intensity colormaps applied to cells in the post-QC Dataset 6 (CODEX) embedding showing high-contrast between immunopositive and immunonegative cell populations cells with the removal of dim outliers (see **Online Supplementary Fig. 10** for full set of UMAP plots colored by marker intensity).

Supplementary Note 2

Developing a Deep Learning (DL) model for automated artifact detection in tissue.

Although tools based on visual review are common in microscopy, there are obvious benefits to machine learning approaches[2-5]. To generate initial training data for a DL model to automatically flag arbitrary artifacts in multiplex IF images, three human annotators assembled ground truth artifact masks for 24 CyCIF channels in 11 serial sections of the same CRC tissue analyzed in this study (Dataset 2, **Supplementary Fig. 1b**). Single channel images (and their corresponding ground truth artifact masks) were cropped into 2048 x 2048-pixel image tiles. After class balancing, the total of 3,787 tiles were split 9:1 into training (3,409) and validation (378) sets. Tissue images differed with respect to the channels that were affected by artifacts (**Supplementary Fig. 3a**). The number of tiles containing artifacts also differed between images, ranging from as many as 463 tiles in image 59 to as few as 129 in image 64 (**Supplementary Fig. 3b**). Of the 3,787 total tiles, 1,734 contained pixels annotated as artifacts (45.8%, **Supplementary Fig. 1c**). Across all tiles, the average percentage of pixels affected by artifacts was ~6.7%.

Our DL model comprised a pretrained ResNet34 encoder[6] coupled to a Feature Pyramid Network (FPN)[7] decoder (ResNet-FPN). Image tiles served as model input and its output was predicted binary artifact masks of the same dimension. To assess the technical reproducibility of artifact predictions, three independent ResNet-FPN models were trained to convergence starting from FPN network weights initialized using different random seeds. Validation loss measured via the Dice Similarity Coefficient ranged from 0.426 to 0.459 (mean = 0.444). To determine the ability of the trained models to generalize across different marker channels, testing was performed on channel 29 of tissue section 54 (**Supplementary Fig. 3d**) which contained artifacts not found in other sections or channels (**Supplementary Fig. 3a**). Performance was assessed by precision-recall (PR) and receiver operating characteristic (ROC) curve analysis. Average precision (AP) ranged from 0.30 to 0.33 for the three models (**Supplementary Fig. 3e**) and area under the ROC curve (AUC) ranged between 0.71 and 0.75 (**Supplementary Fig. 3f**). Predicted artifact masks for all three models were concordant with ground truth annotations (**Supplementary Fig. 3g**). This work demonstrates that the assembly of a DL model for artifact detection in high-plex tissue images is feasible. However, we judge the overall level of performance relative to human reviewers to be inadequate in its current state and strongly suspect that this is due to insufficient training data. CyLinter is nevertheless an ideal way to generate additional training data. Thus, we have established a deposition site at the Synapse data repository (Sage Bionetworks, https://www.synapse.org/#!Synapse:syn24193163/wiki/624232) for collecting CyLinter-curated image artifacts and anticipate that further training of our ResNet-FPN model on this growing corpus of collected artifacts will ultimately yield a highly-performant model for integration into future versions of the CyLinter program.

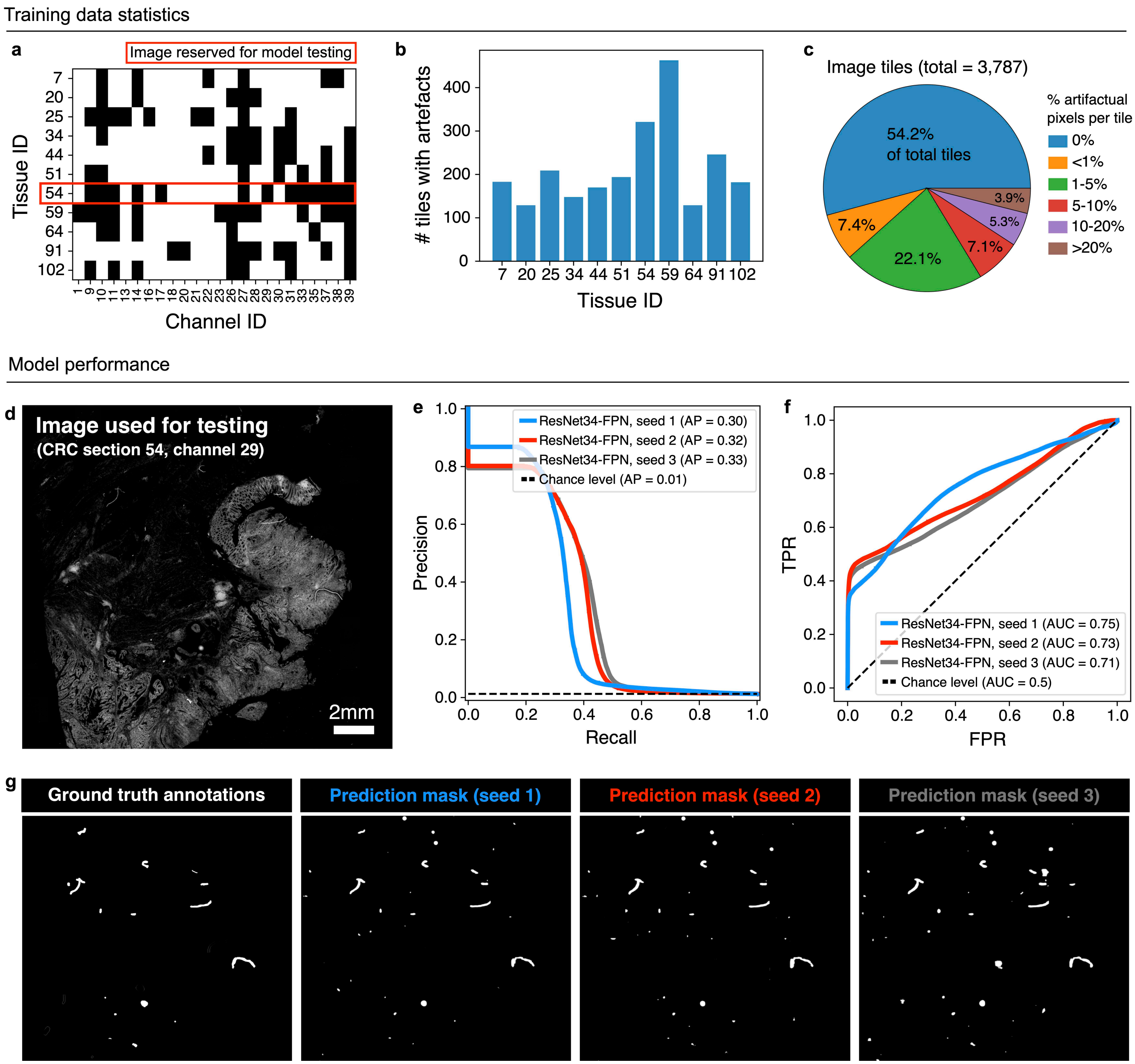

Supplementary Fig. 3 | Developing a DL model for automated artifact detection in tissue. a, Binary matrix showing the channels impacted by visual artifacts (e.g., illumination aberrations, slide debris, etc.) in 11 sections of the same CRC specimen. **b**, Barchart showing the number of 2048 x 2048-pixel image tiles affected by artifacts per tissue section. **c**, Pie chart showing the percentage of total image tiles used for model training and validation (3,787) with different percentages of artifactual pixels (color categories). **d**, Channel 29 of CRC tissue section 54 used for model testing. **e**, Precision-recall plot showing the average precision (AP) for three replicates of the ResNet-FPN model architecture whose FPN networks were initialized with different model weights to evaluate its technical reproducibility. **f**, Receiver operating characteristic (ROC) curve showing the area under the curve (AUC) values for the same three replicates of the ResNet-FPN model shown in panel (e). **g**, Ground truth artifact mask (far left) and predicted artifact masks for the three replicate ResNet-FPN models (blue, red, gray).

Online Supplementary Fig. 1 | Example artifacts in Dataset 1 (TOPACIO) (https://www.synapse.org/#!Synapse:syn53781614). **a**, Twelve (12) examples of tissue folds. **b**, Twelve (12) examples of slide debris. **c**, Four (4) examples of coverslip air bubbles. **d**, Twelve (12) examples of image blur.

Online Supplementary Fig. 2 | Image galleries of clustering cells from pre-QC Dataset 2 (CRC) (https://www.synapse.org/#!Synapse:syn53781627). Twenty (20) examples of cells from each of 22 clusters identified in the pre-QC CRC dataset showing the top three most highly expressed markers (1: green, 2: red, 3: blue) and Hoechst dye (gray). A single white pixel at the center of each image highlights the reference cell. Nuclear segmentation outlines are superimposed to show segmentation quality.

Online Supplementary Fig. 3 | Image galleries of clustering cells from pre-QC Dataset 6 (CODEX) (https://www.synapse.org/#!Synapse:syn53781635). Twenty (20) examples of cells from each of 32 clusters identified in the pre-QC CODEX dataset (normal large intestine, specimen 1) showing the top three highly expressed markers (1: green, 2: red, 3: blue) and Hoechst dye (gray). A single white pixel at the center of each image highlights the reference cell. Nuclear segmentation outlines are superimposed to show segmentation quality.

Online Supplementary Fig. 4 | Image galleries of clustering cells from pre-QC Dataset 1 (TOPACIO) (https://www.synapse.org/#!Synapse:syn53782191). Twenty (20) examples of cells from each of 48 (of 492) clusters identified in the pre-QC TOPACIO dataset showing the top three most highly expressed markers (1: green, 2: red, 3: blue) and Hoechst dye (gray). A single white pixel at the center of each image highlights the reference cell. Nuclear segmentation outlines are superimposed to show segmentation quality.

Online Supplementary Fig. 5 | Image tiles from Dataset 1 (TOPACIO) (https://www.synapse.org/#!Synapse:syn53779745). Down-sampled, single-channel images from the 25 TNBC tissue specimens analyzed in this study used to estimate the number of image tiles impacted by microscopy artifacts. Artifact counts table and patient metadata table are also provided.

Online Supplementary Fig. 6 | Image galleries of clustered cells from post-QC Dataset 2 (CRC) (https://www.synapse.org/#!Synapse:syn53781719). Twenty (20) examples of cells from each of 78 clusters identified in the post-QC CRC dataset showing the top three most highly expressed markers (1: green, 2: red, 3: blue) and Hoechst dye (gray). A single white pixel at the center of each image highlights the reference cell. Nuclear segmentation outlines are superimposed to show segmentation quality.

Online Supplementary Fig. 7 | Image galleries of clustered cells from post-QC Dataset 6 (CODEX) (https://www.synapse.org/#!Synapse:syn53781730). Twenty (20) examples of cells from each of 28 clusters identified in the post-QC CODEX dataset showing the top three most highly expressed markers (1: green, 2: red, 3: blue) and Hoechst dye (gray). A single white pixel at the center of each image highlights the reference cell. Nuclear segmentation outlines are superimposed to show segmentation quality.

Online Supplementary Fig. 8 | Image galleries of clustered cells from post-QC Dataset 1 (TOPACIO) (https://www.synapse.org/#!Synapse:syn53781892). Twenty (20) examples of cells from each of 43 clusters identified in the post-QC TOPACIO dataset showing the top three highly expressed markers (1: green, 2: red, 3: blue) and Hoechst dye (gray). A single white pixel at the center of each image highlights the reference cell. Nuclear segmentation outlines are superimposed to show segmentation quality.

Online Supplementary Fig. 9 | Channel colormaps applied to cells in the pre-QC Dataset 6 (CODEX) embedding (https://www.synapse.org/#!Synapse:syn53781812).

Online Supplementary Fig. 10 | Channel colormaps applied to cells in the post-QC Dataset 6 (CODEX) embedding (https://www.synapse.org/#!Synapse:syn53781819).

A list of centers and principal investigators participating in the Human Tumor Atlas Network (HTAN) can be found here: https://humantumoratlas.org/research-network.

A list of centers and principal investigators participating in the Human BioMolecular Atlas Program (HuBMAP) can be found here: https://hubmapconsortium.org/group-pages-index.

Supplementary References

1. Sternberg, S. R., "Biomedical Image Processing," in Computer, vol. 16, no. 1, pp. 22-34, Jan. 1983, doi: 10.1109/MC.1983.1654163.

2. Prabhakaran, S. et al. Addressing persistent challenges in digital image analysis of cancerous tissues. Preprint at http://biorxiv.org/lookup/doi/10.1101/2023.07.21.548450 (2023).

3. Andhari, M. D. et al. QUAL-IF-AI: quality control of immunofluorescence images using artificial intelligence. Preprint at http://biorxiv.org/lookup/doi/10.1101/2024.01.26.577391 (2024).

4. Ruff, L. et al. A unifying review of deep and shallow anomaly detection. Proc. IEEE 109, 756–795 (2021).

5. Shen, D., Wu, G. & Suk, H.-I. Deep learning in medical image analysis. Annu. Rev. Biomed. Eng. 19, 221–248 (2017).

6. K. He, X. Zhang, S. Ren and J. Sun, "Deep Residual Learning for Image Recognition," 2016 IEEE Conference on Computer Vision and Pattern Recognition (CVPR), Las Vegas, NV, USA, 2016, pp. 770-778, doi: 10.1109/CVPR.2016.90.

7. T. -Y. Lin, P. Dollár, R. Girshick, K. He, B. Hariharan and S. Belongie, "Feature Pyramid Networks for Object Detection," 2017 IEEE Conference on Computer Vision and Pattern Recognition (CVPR), Honolulu, HI, USA, 2017, pp. 936-944, doi: 10.1109/CVPR.2017.106.
