# Supplementary material for: Quality control for single-cell analysis of high-plex tissue profiles using CyLinter
Source: Nat Methods. 2024 Oct 30;21(12):2248–59. doi: 10.1038/s41592-024-02328-0 (PMC11621021; doi:10.1038/s41592-024-02328-0)
Supplement: Supplementary file 2 — Reporting Summary [file 41592_2024_2328_MOESM2_ESM.pdf]

nature portfolio

Corresponding author(s): Peter K. Sorger

Last updated by author(s): May 6, 2024

Reporting Summary

Nature Portfolio wishes to improve the reproducibility of the work that we publish. This form provides structure for consistency and transparency in reporting. For further information on Nature Portfolio policies, see our Editorial Policies and the Editorial Policy Checklist.

Statistics

For all statistical analyses, confirm that the following items are present in the figure legend, table legend, main text, or Methods section.

n/a	Confirmed	
☐	☒	The exact sample size (*n*) for each experimental group/condition, given as a discrete number and unit of measurement
☒	☐	A statement on whether measurements were taken from distinct samples or whether the same sample was measured repeatedly
☐	☒	The statistical test(s) used AND whether they are one- or two-sided<br>*Only common tests should be described solely by name; describe more complex techniques in the Methods section.*
☒	☐	A description of all covariates tested
☒	☐	A description of any assumptions or corrections, such as tests of normality and adjustment for multiple comparisons
☐	☒	A full description of the statistical parameters including central tendency (e.g. means) or other basic estimates (e.g. regression coefficient) AND variation (e.g. standard deviation) or associated estimates of uncertainty (e.g. confidence intervals)
☐	☒	For null hypothesis testing, the test statistic (e.g. *F*, *t*, *r*) with confidence intervals, effect sizes, degrees of freedom and *P* value noted<br>*Give P values as exact values whenever suitable.*
☒	☐	For Bayesian analysis, information on the choice of priors and Markov chain Monte Carlo settings
☒	☐	For hierarchical and complex designs, identification of the appropriate level for tests and full reporting of outcomes
☐	☒	Estimates of effect sizes (e.g. Cohen's *d*, Pearson's *r*), indicating how they were calculated

Our web collection on statistics for biologists contains articles on many of the points above.

Software and code

Policy information about availability of computer code

Data collection

Raw microscopy image tiles (RCPNL files) for the datasets described in this study were processed into stitched, registered, and segmented OME-TIFF files using the MCMICRO image-processing software. Corresponding cell x feature CSV files (i.e., spatial feature tables) were also generated by MCMICRO. Specific algorithms implemented in the MCMICRO image processing pipeline are as follows: BaSiC (v1.0.1) — a Fiji/ImageJ plugin for background and shading correction used to perform flatfield and darkfield image correction; ASHLAR (v1.11.1) — a program for seamless mosaic image processing across imaging cycles; Coreograph (v2.2.0) — a program for dearraying TMA corers into individual TIFF and CSV files (https://github.com/HMS-IDAC/UNetCoreograph); UnMICST (v2.4.7) — an implementation of semantic cell segmentation based on the U-Net deep learning architecture; S3segmenter (v1.2.0) — a watershed algorithm used in conjunction with UnMICST (https://github.com/HMS-IDAC/S3segmenter); MCQuant (v1.3.1) — an algorithm used for per cell feature extraction including X,Y spatial coordinates, segmentation areas, mean marker intensities, and nuclear morphology attributes (https://github.com/labsyspharm/quantification).

Data analysis

The CyLinter software (v0.0.49) described in this study was written in Python3 (versions 3.8 - 3.11) and continually integrates the latest versions of the following data science libraries: napari, magicgui, pyqt, qtpy, pyyaml, tifffile, scikit-image, zarr, pandas, pyarrow, numpy, matplotlib, seaborn, hdbscan, umap-learn, joblib, scikit-learn, scipy, cellcutter, natsort, numba, svglib, and pypdf2. Figure panels shown in this article were generated using combinations of the individual aforementioned data science libraries and the CyLinter program itself. CyLinter source code is freely available under the MIT open-source license at https://labsyspharm.github.io/cylinter/. Scripts used to generate figure panels in this article may be accessed via a dedicated GitHub repository (https://github.com/labsyspharm/cylinter-paper) archived on Zenodo (https://zenodo.org/records/10067803).

For manuscripts utilizing custom algorithms or software that are central to the research but not yet described in published literature, software must be made available to editors and reviewers. We strongly encourage code deposition in a community repository (e.g. GitHub). See the Nature Portfolio guidelines for submitting code & software for further information.

Data

Policy information about availability of data

All manuscripts must include a data availability statement. This statement should provide the following information, where applicable:

- Accession codes, unique identifiers, or web links for publicly available datasets
- A description of any restrictions on data availability
- For clinical datasets or third party data, please ensure that the statement adheres to our policy

This study centers on the analysis of the following seven multiplex imaging datasets: (1) 25 specimens of triple-negative breast cancer from patients enrolled in the TOPACIO clinical trial imaged by CyCIF (ClinicalTrials.gov ID: NCT02657889); (2) a primary human colorectal adenocarcinoma resection imaged by CyCIF (CRC, Lin et al. Cell 2023, PMCID: PMC10019067); (3) a tissue microarray consisting of 123 different healthy and cancerous tissue cores each 1.5 mm in diameter imaged by CyCIF (EMIT TMA22, Synapse: https://www.synapse.org/#!Synapse:syn22345750, Schapiro et al. Nat. Methods 2022, PMCID: PMC8916956); (4) two sections of a single head & neck squamous carcinoma (HNSCC) specimen imaged by CODEX (provided by the laboratory of Kai Wucherpfennig at Dana-Farber Cancer Institute); (5) a whole-slide section of normal human tonsil imaged by mIHC (Synapse: https://www.synapse.org/#!Synapse:syn25174227, Schapiro et al. Nat. Methods 2022, PMCID: PMC8916956); (6) a section of normal human large intestine imaged by CODEX (HuBMAP: https://portal.hubmapconsortium.org/browse/dataset/ae422532f260b3d6fc662aae69b05d33); and (7) a second, independent section of normal human large intestine imaged by CODEX (HuBMAP: https://portal.hubmapconsortium.org/browse/dataset/eaad67a6c6e891ea72cc397c26bd607f). Access to the TOPACIO dataset can be made through the explicit permission of the TOPACIO clinical trial sponsor (Tesaro, Inc.). All other datasets necessary to reproduce the findings in this study can be found at the Sage Bionetworks Synapse data repository at the following URL: https://www.synapse.org/#!Synapse:syn54523217. See Supplementary Table 1 for complete details on dataset identifiers and accession information. All information pertaining to commercial, open-source, or custom code used in the acquisition of previously collected datasets (CRC, EMIT TMA22, HNSCC, tonsil, large intestine) can be found at links to the primary data resources. The TOPACIO dataset was collected during this study using a CyteFinder slide scanning fluorescence microscope and its built-in image acquisition software (RareCyte Inc. Seattle WA).

Research involving human participants, their data, or biological material

Policy information about studies with human participants or human data. See also policy information about sex, gender (identity/presentation), and sexual orientation and race, ethnicity and racism.

Reporting on sex and gender	It is thought that the nature and abundance of microscopy artifacts in images of tissue are not dependent on donor sex or gender. Thus, tissue specimens analyzed in this study were selected without respect to these covariates. Nevertheless, the sex of the donors for the specimens used in this study are as follows: all TOPACIO specimens = female; CRC specimen = male; EMIT TMA22 cores = 44 male/76 female; HNSCC = unknown; tonsil = female; large intestine (samples 1 and 2) = male.
Reporting on race, ethnicity, or other socially relevant groupings	The nature and abundance of microscopy artifacts observed in multiplex images of tissue are thought to be independent of race, ethnicity, or social status of the donor. Thus, these factors were not controlled for in our study. Nevertheless, the race of the donors for the specimens used are as follows: CRC, tonsil, and large intestine (sample 2) = Caucasian; large intestine (sample 1) = African American. The race/ethnicity of other tissue donors is not known.
Population characteristics	Covariate-relevant population characteristics of the human research participants such as age, genotype, past and current diagnosis, and treatment status are not thought to influence the number and quality of microscopy artifacts in multiplex images of tissue. However, limited demographic information was provided and is as follows: Dataset 1 (TOPACIO) comprises tissue from 25 female patients, Dataset 2 (CRC) is from a 69-year-old white male, Dataset3 (EMIT TMA) comprises cores from 44 males and 76 females between the ages of 21 and 86, Dataset 5 is from a 4-year-old female of European ancestry, Dataset 6 is from a 78-year-old African American male, and Dataset 7 is from a 24-year-old white male.
Recruitment	Participants were not recruited for this study.
Ethics oversight	The research described in this study complies with all relevant ethical regulations and was reviewed and approved by the Institutional Review Boards (IRBs) at Brigham and Women's Hospital (BWH), Dana-Farber Cancer Institute (DFCI), and Harvard Medical School (HMS). All patient tissue samples were used after informed written consent.

Note that full information on the approval of the study protocol must also be provided in the manuscript.

Field-specific reporting

Please select the one below that is the best fit for your research. If you are not sure, read the appropriate sections before making your selection.

☒ Life sciences ☐ Behavioural & social sciences ☐ Ecological, evolutionary & environmental sciences

For a reference copy of the document with all sections, see nature.com/documents/nr-reporting-summary-flat.pdf

Life sciences study design

All studies must disclose on these points even when the disclosure is negative.

Sample size	Seven multiplex imaging datasets were used in this study: six whole-slide tissue datasets and a tissue microarray (TMA). Given that this study focuses on the types of artifacts in multiplex tissue imaging and their influence on single-cell data analysis, statistics comparing features within and across populations that would otherwise require computing sample sizes were not performed. However, we believe that the tissue areas

	of the 6 whole-slide Datasets (ranging from 6-353 mm2) and the 123 TMA cores (~2 mm2 each) comprising Dataset 3 together provide sufficient material to robustly survey recurrent artifacts in multiplex imaging data.
Data exclusions	Lack of antibody labeling, use of secondary antibodies alone for the purpose of tissue blocking, poor tissue quality during latter imaging cycles, and immunomarker redundancy precluded the use of certain channels in the multiplex images described in this study. The following channels were excluded from each dataset:<br><br>TOPACIO: anti-Rat (secondary only), anti-rabbit (secondary only), anti-Goat (secondary only), pSTAT1, Ki67, DNA (cycle 8), STING, pTBK1, pSTAT3, DNA (cycle 9), PCNA, HLAA, and cPARP.<br><br>CRC: AF488 (secondary only), AF555 (secondary only), AF647(secondary only), A488 (secondary only), A555 (secondary only), A647 (secondary only), anti_NaKATPase, Ki67_488, Ki67_570.<br><br>EMIT TMA22: Rabbit IgG (secondary only), Goat IgG (secondary only), Mouse IgG (secondary only), CD56, CD13, pAUR, CCNE, CDKN2A, PCNA_1, CDKN1B_2.<br><br>HNSCC: empty_ch2_cycle1, empty_ch3_cycle1, empty_ch4_cycle1, empty_ch2_cycle4, empty_ch4_cycle4, empty_ch2_cycle5, empty_ch2_cycle6, empty_ch2_cycle8, empty_ch2_cycle9, empty_ch3_cycle9, empty_ch4_cycle9.<br><br>Normal tonsil: no channels were excluded from this dataset.<br><br>Normal large intestine (sample 1): DRAQ5 (excluded due to its redundancy with Hoechst).<br><br>Normal large intestine (sample 2): DRAQ5 (excluded due to its redundancy with Hoechst).
Replication	To verify the reproducibility of the findings in this study, we performed in depth analysis of microscopy artifacts and their influence on derived single-cell data across seven different high-plex imaging datasets in the form of whole-slide images and a tissue microarray acquired via one of three different imaging technologies: CyCIF, CODEX, and mIHC. Similar artifacts were observed in all datasets regardless of the technology used to acquire the images and confounded data analysis and interpretation to the same extent.
Randomization	Randomization was not performed in this study, as groups of tissues were not compared.
Blinding	Blinding was not performed in this retrospective and non-interventional study, as groups of tissues were not compared.

Reporting for specific materials, systems and methods

We require information from authors about some types of materials, experimental systems and methods used in many studies. Here, indicate whether each material, system or method listed is relevant to your study. If you are not sure if a list item applies to your research, read the appropriate section before selecting a response.

Materials & experimental systems

n/a	Involved in the study	
☐	☒	Antibodies
☒	☐	Eukaryotic cell lines
☒	☐	Palaeontology and archaeology
☒	☐	Animals and other organisms
☒	☐	Clinical data
☒	☐	Dual use research of concern
☒	☐	Plants

Methods

n/a	Involved in the study	
☒	☐	ChIP-seq
☒	☐	Flow cytometry
☒	☐	MRI-based neuroimaging

Antibodies

Antibodies used	The following antibodies were used in the acquisition of the TOPACIO dataset (Name, Clone, Vendor, Catalog number, RRID, dilution):<br><br>Donkey anti-Rat A488 (secondary only), polyclonal, Invitrogen, A21208, AB_2535794, 1:1000<br>Donkey anti-Rabbit A555 (secondary only), polyclonal, Invitrogen, A31572, AB_162543, 1:1000<br>Donkey anti-Goat A647 (secondary only), polyclonal, Invitrogen, A21447, AB_2535864, 1:1000<br>CD3 (secondary conjugated), CD3-12, Abcam, ab11089, AB_2889189, 1:200<br>PD-L1 (secondart conjugated), E1L3N, Cell Signaling Technology, 13684S, AB_2687655, 1:200<br>53BP1 (secondary conjugated), polyclonal, Bethyl Laboratories, A303-906A, AB_2620256, 1:200<br>E-Cadherin(A488), 24E10, Cell Signaling Technology, 3199S, AB_2291471, 1:400<br>panCK(e570), AE1/AE3, EBioscience, 41-9003-82, AB_11218704, 1:800<br>PD-1(A647), EPR4877(2), abcam, ab201825, AB_2728811, 1:200<br>CD8a(A488), AMC908, EBioscience, 53-0008-82, AB_2574413, 1:200<br>CD45(PE), 2D1, R&D, FAB1430P, AB_2237898, 1:100<br>GrB(A647), 2C5, Santa Cruz, sc-8022AF647, AB_2232723, 1:200<br>CD163(A488), EPR14643-36, Abcam, ab218293, AB_2889155, 1:400<br>CD68(PE), D4B9C, Cell Signaling Technology, 79594S, AB_2799935, 1:200

CD20(e660), L26, EBioscience, 50-0202-80, AB_11151691, 1:400
CD4(A488), polyclonal, R&D Systems, FAB8165G, AB_2728839, 1:200
FOXP3(e570), 236A/E7, EBioscience, 41-4777-82, AB_2573609, 1:100
SMA(e660), 1A4, EBioscience, 50-9760-82, AB_2574362, 1:800
CD11b(A488), C67F154, EBioscience, 53-0196-82, AB_2637196, 1:150
pSTAT1(A555), 58D6, Cell Signaling Technology, 8183S, AB_10860600, 1:200
yH2AX(A647), 2F3, Biolegend, 613407, AB_2295046, 1:200
CD57(FITC), NK-1, BD, 561906, AB_395986, 1:100
Ki67(e570), 20Raj1, EBioscience, 41-5699-82, AB_11220278, 1:100
MHCII/HLA-DPB1(A647), EPR11226, Abcam, ab201347, AB_2861375, 1:400
STING(A488), EPR13130, Abcam, ab198950, AB_2889208, 1:400
pTBK1(A555), D52C2, Cell Signaling Technology, 13498S, AB_2943237, 1:200
pSTAT3(A647), D3A7, Cell Signaling Technology, 4324S, AB_10694637, 1:200
PCNA(A488), PC10, Cell Signaling Technology, 8580S, AB_2617115, 1:400
HLA-A(A555), EP1395Y, Abcam, ab207872, AB_2889202, 1:400
cPARP(A647), D64E10, Cell Signaling Technology, 6987S, AB_10699459, 1:100

Validation

The performance of antibodies used in the collection of the TOPACIO dataset has been previously validated in prior publications (PMIDs: 29993362, 31534232, 30388455) and is indexed on the landing page for recommended CycIF antibodies at https://www.cycif.org/antibodies/recommended. The performance of these antibodies in the current study was confirmed through visual inspection of staining intensity and pattern by board-certified pathologists on tissues known to express their target antigens. Additional validation information on the commercially-available antibodies listed in the "Antibodies Used" subsection above can be found in their associated data product sheets at the manufacture's website.

Plants

Seed stocks

Report on the source of all seed stocks or other plant material used. If applicable, state the seed stock centre and catalogue number. If plant specimens were collected from the field, describe the collection location, date and sampling procedures.

Novel plant genotypes

Describe the methods by which all novel plant genotypes were produced. This includes those generated by transgenic approaches, gene editing, chemical/radiation-based mutagenesis and hybridization. For transgenic lines, describe the transformation method, the number of independent lines analyzed and the generation upon which experiments were performed. For gene-edited lines, describe the editor used, the endogenous sequence targeted for editing, the targeting guide RNA sequence (if applicable) and how the editor was applied.

Authentication

Describe any authentication procedures for each seed stock used or novel genotype generated. Describe any experiments used to assess the effect of a mutation and, where applicable, how potential secondary effects (e.g. second site T-DNA insertions, mosiacism, off-target gene editing) were examined.
